# Hippocampal Glutamate, Resting Perfusion and the Effects of Cannabidiol in Psychosis Risk

**DOI:** 10.1093/schizbullopen/sgad022

**Published:** 2023-08-14

**Authors:** Cathy Davies, Matthijs G Bossong, Daniel Martins, Robin Wilson, Elizabeth Appiah-Kusi, Grace Blest-Hopley, Paul Allen, Fernando Zelaya, David J Lythgoe, Michael Brammer, Jesus Perez, Philip McGuire, Sagnik Bhattacharyya

**Affiliations:** Department of Psychosis Studies, Institute of Psychiatry, Psychology and Neuroscience, King’s College London, London, UK; Department of Neuroimaging, Institute of Psychiatry, Psychology and Neuroscience, King’s College London, London, UK; Department of Psychiatry, University Medical Center Utrecht Brain Center, Utrecht University, Utrecht, The Netherlands; Department of Neuroimaging, Institute of Psychiatry, Psychology and Neuroscience, King’s College London, London, UK; National Institute for Health Research (NIHR) Maudsley Biomedical Research Centre (BRC), South London and Maudsley NHS Foundation Trust, London, UK; Department of Psychosis Studies, Institute of Psychiatry, Psychology and Neuroscience, King’s College London, London, UK; Department of Psychosis Studies, Institute of Psychiatry, Psychology and Neuroscience, King’s College London, London, UK; Department of Psychosis Studies, Institute of Psychiatry, Psychology and Neuroscience, King’s College London, London, UK; Department of Psychosis Studies, Institute of Psychiatry, Psychology and Neuroscience, King’s College London, London, UK; Department of Neuroimaging, Institute of Psychiatry, Psychology and Neuroscience, King’s College London, London, UK; Department of Neuroimaging, Institute of Psychiatry, Psychology and Neuroscience, King’s College London, London, UK; Department of Neuroimaging, Institute of Psychiatry, Psychology and Neuroscience, King’s College London, London, UK; Department of Neuroimaging, Institute of Psychiatry, Psychology and Neuroscience, King’s College London, London, UK; CAMEO Early Intervention Service, Cambridgeshire and Peterborough NHS Foundation Trust, Cambridge, UK; Institute of Biomedical Research (IBSAL), Department of Medicine, Universidad de Salamanca, Salamanca, Spain; Department of Psychiatry, University of Oxford, Oxford, UK; NIHR Oxford Health Biomedical Research Centre, Oxford, UK; Oxford Health NHS Foundation Trust, Oxford, UK; Department of Psychosis Studies, Institute of Psychiatry, Psychology and Neuroscience, King’s College London, London, UK

**Keywords:** magnetic resonance spectroscopy, clinical high risk for psychosis, at-risk mental state, cerebral blood flow

## Abstract

**Background:**

Preclinical and human data suggest that psychosis onset involves hippocampal glutamatergic dysfunction, driving hyperactivity and hyperperfusion in a hippocampal-midbrain-striatal circuit. Whether glutamatergic dysfunction is related to cerebral perfusion in patients at clinical high risk (CHR) for psychosis, and whether cannabidiol (CBD) has ameliorative effects on glutamate or its relationship with perfusion remains unknown.

**Methods:**

Using a double-blind, parallel-group design, 33 CHR patients were randomized to a single 600 mg dose of CBD or placebo; 19 healthy controls did not receive any drug. Proton magnetic resonance spectroscopy was used to measure glutamate concentrations in left hippocampus. We examined differences relating to CHR status (controls vs placebo), effects of CBD (placebo vs CBD), and linear between-group effects, such that placebo>CBD>controls or controls>CBD>placebo. We also examined group × glutamate × cerebral perfusion (measured using Arterial Spin Labeling) interactions.

**Results:**

Compared to controls, CHR-placebo patients had significantly lower hippocampal glutamate (*P* =.015) and a significant linear relationship was observed across groups, such that glutamate was highest in controls, lowest in CHR-placebo, and intermediate in CHR-CBD (*P* =.031). Moreover, there was a significant interaction between group (controls vs CHR-placebo), hippocampal glutamate, and perfusion in the putamen and insula (*P*_FWE_ =.012), with a strong positive correlation in CHR-placebo vs a negative correlation in controls.

**Conclusions:**

Our findings suggest that hippocampal glutamate is lower in CHR patients and may be partially normalized by a single dose of CBD. Furthermore, we provide the first in vivo evidence of an abnormal relationship between hippocampal glutamate and perfusion in the striatum and insula in CHR.

## Introduction

Preclinical and human data suggest that the onset of psychosis involves hippocampal glutamatergic dysfunction, driving hyperactivity and hyperperfusion in a hippocampal-midbrain-striatal circuit.^1–3^ Specifically, preclinical models demonstrate that NMDA receptor hypofunction on GABAergic interneurons leads to elevated hippocampal glutamate and hypermetabolism.^3^ In turn, hippocampal glutamatergic pyramidal cell disinhibition is thought to lead to excess excitatory drive in projections to the midbrain-striatum, causing hyper-responsivity of midbrain dopamine neurons,^4^ striatal hyperdopaminergia and the emergence of psychotic-like phenotypes (supplementary figure S1).^1,3–5^ Consistent with this, evidence from human studies suggests that across the psychosis continuum, patients have altered concentrations of hippocampal glutamate or Glx (a composite of glutamate and glutamine),^6,7^ hippocampal and striatal hyperperfusion^3,8–11^ and elevated striatal dopamine synthesis capacity (supplementary figure S1).^12,13^ Importantly, these pathophysiological features appear to emerge prior to the onset of psychosis in people at clinical high risk (CHR), progressively worsening and/or spreading as they transition to full-blown psychosis.^2,3,9,13^

In CHR individuals, previous research has identified abnormal concentrations of hippocampal glutamate and/or Glx, although in contrast to the more consistent increases found in established psychosis,^6,7,14–16^ both increases,^17^ decreases^18,19^ and often no differences^20–23^—including at the meta-analytic level^24,25^—have been reported. Nevertheless, within CHR groups, hippocampal glutamate is greater in those with poor vs good outcomes^23^ and (Glx) may be related to symptom severity.^26^ Separately, elevated perfusion (or cerebral blood flow; CBF) has been observed in the hippocampus,^8,10^ striatum,^8,27^ and prefrontal cortex^8^ in CHR patients, and there is some evidence that this may be associated with altered neurochemistry. For example, previous work has linked prefrontal GABA levels^28^ and striatal dopamine function^29^ to hippocampal perfusion in CHR patients, and anterior cingulate glutamate/Glx to hippocampal perfusion in people with high schizotypy.^30^ Although differences in hippocampal glutamate and regional CBF have been identified (separately) in prior CHR studies, whether and how these parameters are associated with each other, and whether any such relationship is abnormal in CHR patients, has yet to be directly tested. A deeper understanding of how hippocampal glutamatergic dysfunction is related to other pathophysiological features—particularly within the hippocampal-midbrain-striatal circuit—would enhance understanding of the mechanisms underlying psychosis risk and may illuminate novel targets for preventative treatments. Given the current lack of effective pharmacotherapies for CHR patients,^31,32^ this remains a critical research priority.

One of the most promising candidate treatments is cannabidiol (CBD), a phytocannabinoid constituent of the cannabis plant.^33^ In contrast to the psychotomimetic and potential anxiogenic effects^34–38^ of delta-9-tetrahydrocannabinol, the main intoxicating cannabinoid in cannabis, CBD is non-intoxicating and has anxiolytic^39,40^ and antipsychotic properties.^41–43^ CBD modulates brain activation in response to cognitive and emotional fMRI tasks, particularly in medial temporal cortex and striatal regions, in both healthy and established psychosis cohorts.^44–49^ In CHR patients, we previously demonstrated that a 600 mg dose of CBD partially normalizes hippocampal resting perfusion^50^ and mediotemporal and striatal function during various fMRI tasks,^51,52^ such that perfusion/activation in the CBD group was intermediate between that of healthy controls and CHR patients under placebo. Accumulating evidence further suggests that CBD may have effects on glutamate. In people with first-episode psychosis, we previously found that CBD increased hippocampal glutamate, an effect linked to the greater reduction of positive symptoms observed under its influence.^53^ Independent work shows that CBD modulates Glx in basal ganglia and prefrontal cortex across ASD and neurotypical individuals.^54^ Altogether, these findings suggest that CBD may have effects on glutamate and cerebral blood flow in humans, two pathophysiological features strongly implicated in psychosis onset. However, whether CBD can normalize glutamatergic dysfunction (or its relationship with blood flow) in CHR patients is yet to be examined.

To fill this gap in knowledge, we examined hippocampal glutamatergic dysfunction in the CHR state and the effects of CBD using Proton Magnetic Resonance Spectroscopy (^1^H-MRS) and three parallel groups: CHR patients randomized to a single oral 600 mg dose of CBD or placebo and healthy controls. We first established whether hippocampal glutamate levels are altered in CHR-placebo patients relative to controls. We then tested our primary hypothesis that CBD would at least partially normalize alterations in glutamate levels, such that a significant linear relationship (placebo>CBD>controls, or controls>CBD>placebo) would exist across groups. Finally, to probe the broader mechanistic relevance of glutamatergic dysfunction, we examined whether the relationship between glutamate and regional CBF (measured using whole-brain Arterial Spin Labeling; ASL) differed between groups and assessed the effects of CBD on this interaction.

## Methods

### Participants

The study (ISRCTN46322781) received Research Ethics (Camberwell St Giles) approval and all participants provided written informed consent. Thirty-three antipsychotic-naive CHR^55^ individuals, aged 18–35, were recruited from early detection services in the United Kingdom (supplementary methods). Nineteen age (within 3 years), sex, and ethnicity-matched healthy controls were recruited locally. Exclusion criteria included history of psychotic or manic episodes, current DSM-IV diagnosis of substance dependence, IQ < 70, neurological disorder, and contraindication to MRI or CBD. Participants were required to abstain from cannabis for 96 hours (supplementary material), other recreational substances for 2 weeks, alcohol for 24 hours, and caffeine/nicotine for 6 hours before attending. Urine drug screening was conducted prior to scanning.

### Design, Materials, and Procedure

Using a randomized, double-blind, placebo-controlled, 3-arm parallel-group design, CHR participants were randomized to a single oral 600 mg dose of CBD (THC-Pharm, Germany) or a matched placebo capsule. Psychopathology was measured at baseline (before drug administration) using the Comprehensive Assessment of At-Risk Mental States^55^ and State-Trait Anxiety Inventory-State Subscale.^56^ Following a standard light breakfast, participants were administered the capsule of CBD or placebo (at ~11 AM) and 180 minutes later, underwent a battery of MRI sequences. Healthy control participants were investigated under identical conditions but did not receive any drug. Plasma CBD levels were sampled at baseline and at 120 and 300 minutes after drug administration.

### Magnetic Resonance Imaging

**Fig. 1.**
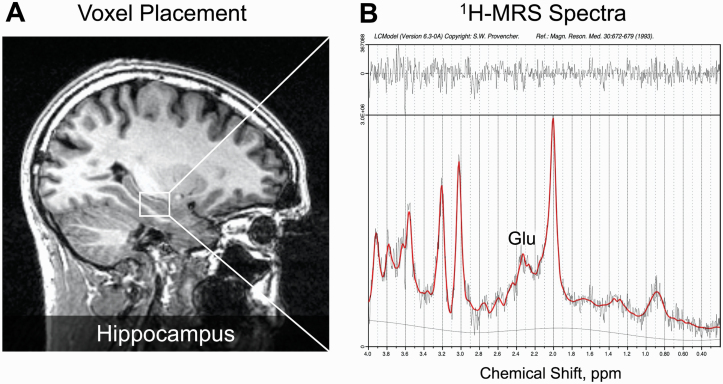
Illustrative example of ^1^H-MRS voxel positioning and spectra in left hippocampus. In panel (A), example voxel placement in the left hippocampus is indicated by the box. Panel (B) shows the ^1^H-MRS spectrum obtained (black line) from the voxel in A and the overlay of the spectral fit (red line). Glu indicates glutamate and ppm, parts per million.

All scans were conducted on a General Electric Signa HDx 3T MR system with an 8-channel head coil. A whole-brain 3D sagittal T1-weighted scan (TE = 2.85ms; TR = 6.98 ms; TI = 400 ms; flip angle = 11°, voxel size = 1.0 × 1.0 × 1.2 mm) was acquired for voxel planning, coregistration and spatial normalization of the ASL data, and calculation of ^1^H-MRS voxel tissue content. ^1^H-MRS spectra were acquired in the left hippocampus (figure 1) using conventional Point-Resolved Spectroscopy acquisition (PRESS; TR = 3000 ms; TE = 30 ms; 96 averages) in a 6-minute scan. We employed the standard GE PROBE (Proton Brain Examination) sequence, which uses a standardized, chemically selective suppression (CHESS) water suppression routine. Unsuppressed water reference spectra (16 averages) were also acquired as part of the standard acquisition for subsequent eddy current correction and water scaling. Shimming was optimized, with auto-prescan performed twice before each scan. Using standardized protocols, the hippocampal voxel (right–left, anterior–posterior, superior–inferior: 20 × 20 × 15 mm) was prescribed from the structural T1-weighted scan, positioned over the center of the left hippocampus as consistently as possible (across subjects) by experienced radiographers. The voxel size was fixed across participants. Structural T1-weighted images were segmented using Statistical Parametric Mapping (SPM8) to enable calculation and correction for ^1^H-MRS voxel tissue content (supplementary methods). CBF was measured using 3D pseudo-Continuous ASL with acquisition parameters and preprocessing procedures in line with previous studies, as detailed in supplementary methods.

### ^1^H-MRS Data Processing

Spectra were analyzed using LCModel/6.3-0A^57^ using the standard basis set of 16 metabolites (supplementary methods). Poorly fitted metabolite peaks (Cramer-Rao minimum variance bounds [CRLB] > 20% as reported by LCModel) were excluded from further analysis. Water-scaled glutamate (primary outcome), glutamate plus glutamine (Glx), myo-inositol, creatine, choline, and N-acetylaspartate values were corrected for voxel tissue composition (supplementary methods). Spectral quality was further assessed using signal-to-noise ratio and spectral linewidths (full width at half-maximum; FWHM).

### Statistical Analysis

Statistical analyses of ^1^H-MRS and other non-imaging data were performed in SPSS/27. Pairwise differences in clinical and demographic variables were examined using independent *t*-tests for continuous data and chi-square tests for categorical data. Potential group differences in data quality, including FWHM, signal-to-noise ratio, CRLB, and voxel tissue proportions were examined using independent *t*-tests (pairwise), and one-way ANOVA for any differences between groups. Before testing our primary hypothesis, we used independent samples *t*-tests to first establish (1) whether hippocampal glutamate levels were altered in the CHR-placebo group vs controls, and (2) whether CHR patients under a single dose of CBD had altered hippocampal glutamate levels compared to CHR patients under placebo. Then, to test our primary hypothesis that glutamate levels in the CBD-treated group would be intermediate between that of the healthy control and placebo-treated group, we examined whether a linear relationship (controls>CBD>placebo; or placebo>CBD>controls) existed across groups, using one-way ANOVA and unweighted polynomial contrasts for linear degree. Linear trend (ie, relationship) analyses are distinct from standard ANOVA in that they directly test for specific relationships (such as linear or quadratic) across group means, which are not tested by the standard *F*-test.^58^ In the case of unequal variances, the same ANOVA was run using manual group weights (coefficients −1, 0, 1) which provides statistics (*t*-distribution) without assuming equal variances. Exploratory analyses of the secondary metabolites (Glx, myo-inositol, creatine, choline, and N-acetylaspartate) were conducted using the same tests (described above) as for glutamate. The influence of individual metabolite values whose corresponding z-scores were >3 or <−3 (which may indicate outliers) were investigated by rerunning statistical tests without them in sensitivity analyses. Significance was set at *P* < .05 (2-tailed) and effect sizes were reported as Cohen’s d.

### Glutamate × CBF × Group Interactions

Group differences in the relationship between hippocampal glutamate and cerebral blood flow (glutamate × CBF × group interactions) were analyzed using SPM12 in Matlab/R2018b. Using CBF as the dependent variable, glutamate levels were entered as a covariate of interest in independent *t*-tests for the 2 pairwise contrasts (placebo vs control; CBD vs placebo), and a flexible factorial ANCOVA model for the linear between-group analyses. In line with previous CHR studies of CBF,^8,10^ mean-centered age, sex, smoking status, and years of education (the latter included due to significant group differences in our sample), as well as mean global CBF, were entered as nuisance covariates. We conducted a whole-brain search using an explicit gray matter mask (MNI152, thresholded at >.50) and cluster-level inference (cluster-forming threshold *P* < .005; clusters reported as significant at *P* < .05 using FWE cluster correction in SPM).

## Results

There were no between-group differences in the majority of demographic and baseline clinical characteristics, except for fewer years of education in the placebo group relative to controls (table 1), as previously reported.^51,52,59^ None of the patients were currently taking antidepressants, anxiolytics or mood stabilizers, and all were antipsychotic naïve. Five CHR patients (15%) had a self-reported previous history of depression and one (3%) had a history of depression and anxiety. In the CBD group, mean plasma CBD levels were 126.4 nM (*SD* = 221.8) and 823.0 nM (*SD* = 881.5) at 120 and 300 minutes after drug intake, respectively. ^1^H-MRS data were available for all participants. ASL data were available for *n* = 14 in the placebo group, *n* = 14 in the CBD group, and *n* = 19 healthy controls.

**TABLE 1.** Sociodemographic and Clinical Characteristics at Baseline

Characteristic	CBD (n = 16)	Placebo (n = 17)	Control (n = 19)^1^	Pairwise Comparison	
				Control vs Placebo	Placebo vs CBD
Age, years; mean (SD)	22.7 (5.08)	24.1 (4.48)	23.9 (4.15)	p=.91^2^	p=.42^2^
Sex, N (%) male	10 (63)	7 (41)	11 (58)	p=.32^3^	p=.22^3^
Ethnicity, N (%)					
White	10 (63)	7 (41)	11 (58)	p=.59^3^	p=.43^3^
Black	2 (13)	5 (29)	5 (26)		
Asian	0 (0)	1 (6)	0 (0)		
Mixed	4 (25)	4 (24)	3 (16)		
Education, years; mean (SD)	14.4 (2.71)	12.6 (2.76)	16.9 (1.58)	**p<.001** ^**2**^	p=.06^2^
Handedness, N (%) right	14 (88)	17 (100)	18 (95)	p=.37^3^	p=.16^3^
CAARMS Positive, mean (SD)	40.19 (20.80)	42.94 (29.47)	NA	NA	p=.76^2^
CAARMS Negative, mean (SD)	23.25 (16.49)	28.41 (20.49)	NA	NA	p=.43^2^
CHR Subtype^6^, N APS/BLIPS/GRD	13/1/2	13 / 0 / 4	NA	NA	p=.44^3^
Transition to psychosis^7^, N yes	2	4	NA	NA	NA
STAI-S, mean (SD)	40.31 (9.07)	38.94 (10.18)	NA	NA	p=.69^2^
Urine drug screen results, N (%)					
Clean	10 (63)	8 (47)	NA^4^	NC^1^	p=.45^3^
THC	2 (13)	5 (29)	NA^4^		
Morphine	1 (6)	0 (0)	NA^4^		
Benzodiazepines	0 (0)	1 (6)	NA^4^		
PCP	0 (0)	1 (6)	NA^4^		
Missing	3 (19)	2 (12)	NA^4^		
Current nicotine use, N (%) yes	9 (56)	5 (29)	2 (11)	p=.15^3^	p=.12^3^
Current alcohol use, N (%) yes	11 (69)	10 (59)	NA	NC^1^	p=.59^3^
Lifetime cannabis use, N (%) yes	15 (94)	17 (100)	NA^5^	NC^1^	p=.48^3^
Current cannabis use, N (%) yes	7 (44)	7 (41)	NA^5^	NC	p=.88^3^
Cannabis use frequency, N current users (% total group)^8^					
More than once a week	5 (31)	5 (29)	NA	NC^1^	p=.14^3^
Once/twice monthly	0 (0)	2 (12)	NA		
Few times a year	2 (13)	0 (0)	NA		
Only once/twice lifetime	0 (0)	0 (0)	NA		

*Abbreviations:* CAARMS, Comprehensive Assessment of At-Risk Mental States; CBD, cannabidiol; CHR, Clinical High Risk for Psychosis; N, number of subjects; NA, not applicable; NC, not compared^1^; PCP, phencyclidine; STAI-S, State-Trait Anxiety Inventory-State Subscale; THC, Δ9-tetrahydrocannabinol. Bold text indicates significant difference (p<.05).

^1^Controls were selected to have minimal drug use and hence were not compared with CHR participants on these parameters;

^2^Independent t-test;

^3^Pearson chi-squared test;

^4^Controls tested negative on urine drug screen for all substances tested;

^5^Cannabis use less than 10 times lifetime (no current users);

^6^CAARMS subgroup: BLIPS brief limited intermittent psychotic symptoms, APS attenuated psychotic symptoms, GRD genetic risk and deterioration;

^7^Data on later transition to psychosis was not systematically collected and thus these numbers should be interpreted with caution, particularly as transition is a time-dependent outcome;

^8^The count data (N) represent the number of current cannabis users (of which there were 7 in each CHR group) who reported typical cannabis use at each given frequency. The p-value shown relates to this count data. Percentages reflect the % of the total group sample who are current cannabis users and typically using at each frequency.

### ^1^H-MRS Data Quality

Representative spectra for the hippocampal voxel are provided in figure 1. Spectra were of good quality: Aside from omission of choline data for 2 CHR subjects (see table 2) due to CRLB > 20%, no glutamate or other metabolite data were excluded. No significant differences in spectral quality nor voxel tissue content were observed between groups (table 2). All metabolite values fell within a z-score of +/− 3.

**Table 2.** Spectral and Structural Voxel Data. Mean ± SD Estimates of Linewidths, Signal-to-Noise Ratios, CRLB, and Voxel Proportions of White Matter, Gray Matter, and CSF in the Hippocampus Across the 3 Groups

	Control (*n* = 19)	Placebo (*n* = 17)	CBD (*n* = 16)	Control vs Placebo	Placebo vs CBD	ANOVA
*Spectral and structural voxel data*						
FWHM	0.07 ± 0.02	0.07 ± 0.01	0.07 ± 0.01	*P =* .84	*P =* .18	*P =* .50
SNR	12.53 ± 2.57	13.53 ± 2.40	13.38 ± 2.13	*P =* .24	*P =* .85	*P =* .40
WM	0.33 ± 0.07	0.33 ± 0.06	0.36 ± 0.07	*P =* .94	*P =* .16	*P =* .27
GM	0.63 ± 0.06	0.63 ± 0.05	0.61 ± 0.07	*P =* .96	*P =* .30	*P =* .48
CSF	0.04 ± 0.02	0.04 ± 0.01	0.03 ± 0.02	*P =* .63	*P =* .09	*P =* .07
*Cramér Rao Lower Bounds (CRLB)*						
Glu	9.53 ± 2.06	9.76 ± 1.68	9.81 ± 2.61	*P =* .71	*P =* .95	*P =* .91 ^b^
Glx	10.26 ± 2.10	10.29 ± 2.14	10.38 ± 3.30	*P =* .97	*P =* .93	*P =* .99 ^b^
NAA	4.21 ± 1.32	3.88 ± 0.70	3.50 ± 0.63	*P =* .35	*P =* .11	*P =* .09 ^b^
Cho^a^	4.00 ± 1.00	3.94 ± 0.77	3.93 ± 0.80	*P =* .84	*P =* .99	*P =* .97
ml	5.58 ± 1.22	5.76 ± 1.20	5.94 ± 2.11	*P =* .65	*P =* .77	*P =* .79
Cre	3.89 ± 0.66	4.06 ± 0.66	3.88 ± 0.72	*P =* .46	*P =* .45	*P =* .69

*Abbreviations:* FWHM, full width at half-maximum (linewidth) in ppm (parts per million); WM, white matter; GM, gray matter; CSF, cerebrospinal fluid; CRLB, Cramér Rao Lower Bounds; SNR, signal-to-noise ratio; Glu, Glutamate; Glx, Glutamate + Glutamine; NAA, N-acetylaspartate; Cho, Choline; ml, myo-Inositol; Cre, Creatine.

^a^Choline data for 2 CHR subjects (one from each of the CBD and placebo groups) was omitted due to CRLB >20%;

^b^Welch’s test due to inhomogeneity of variances.

### Hippocampal Glutamate – Pairwise Effects of CHR Status and CBD

**Fig. 2.**
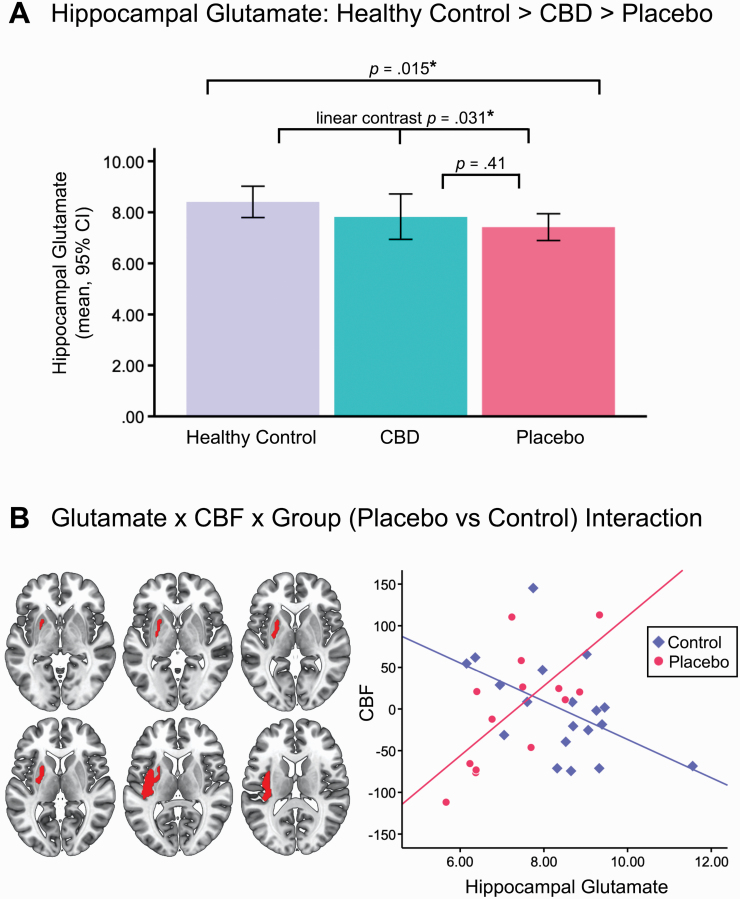
(A) Hippocampal glutamate levels across the three groups. CHR patients in the placebo group had significantly lower glutamate relative to healthy controls (*P =* .015) and there was a significant linear relationship across groups (such that controls > CBD > placebo; *P =* .031). In the left panel of (B), axial sections showing the significant cluster (in red) identified in the glutamate × CBF × group (placebo vs control) interaction analyses (peak MNI X/Y/Z = −38/−12/10, *T*(24) = 5.63, *k* = 679, *P*_FWE_ = .012). The left side of the brain is shown on the left of the images. In the right panel of (B), scatterplot depicting the relationship between CBF (in the putamen-insula cluster shown in the left panel) and hippocampal glutamate by group. This post hoc analysis was used to determine the direction of the significant interaction: Covariate-adjusted CBF values (mean CBF over all voxels, arbitrary units) were extracted from the significant cluster (left panel) for each subject from the T-contrast using the MarsBaR toolbox in SPM.

Compared to healthy controls, CHR patients in the placebo group had significantly lower hippocampal glutamate (mean ± SD in controls = 8.41 ± 1.27; placebo = 7.42 ± 1.02; *t*(34)= 2.55, *P* = .015, *d* = 0.85) (figure 2A, table 3). Although hippocampal glutamate levels were numerically higher in the CBD (7.83 ± 1.67) vs placebo (7.42 ± 1.02) group, the pairwise difference was not significant (*P* = .41, *d* = 0.30; table 3).

**Table 3.** Tissue-Corrected Metabolite Values in the Hippocampus (mean ± SD) Across the 3 Groups, With Pairwise Comparisons (Healthy Control vs Placebo; Placebo vs CBD) and 3-Way Contrasts for a Between-Group Linear Relationship (Controls > CBD > Placebo, or Placebo > CBD > Controls)

Metabolites	Control (*n* = 19)	Placebo (*n* = 17)	CBD (*n* = 16)	Group Comparisons		
				Control vs Placebo	Placebo vs CBD	ANOVA Linear Contrast
*A priori*						
Glu	8.41 ± 1.27	7.42 ± 1.02	7.83 ± 1.67	*t*(34) = 2.55, *P =* .015 *	*t*(24.59) = 0.85, *P =* .41	*F*(1,49) = 4.91, *P =* .031 *
*Exploratory*						
Glx	11.32 ± 1.79	10.35 ± 1.60	10.92 ± 3.02	*t*(34) = 1.70, *P =* .098	*t*(22.50) = 0.66, *P =* .51	*t*(34) = 1.71, *P =* .096 ^b^
NAA	9.52 ± 0.77	8.96 ± 1.07	8.89 ± 0.90	*t*(34) = 1.84, *P =* .074	*t*(31) = 0.18, *P =* .86	*F*(1,49) = 3.44, *P =* .070
Cho ^a^	2.44 ± 0.26	2.26 ± 0.41^a^	2.27 ± 0.44^a^	*t*(24.4) = 1.49, *P =* .15	*t*(29) = 0.05, *P =* .96	*F*(1,47) = 1.94, *P =* .17
ml	6.69 ± 1.01	5.86 ± 1.01	6.09 ± 1.45	*t*(34) = 2.46, *P =* .019 *	*t*(31) = 0.54, *P =* .59	*F*(1,49) = 4.58, *P =* .037 *
Cre	7.61 ± 0.77	6.92 ± 1.32	7.19 ± 1.36	*t*(25.28) = 1.88, *P =* .072	*t*(31) = 0.59, *P =* .56	*t*(25.28) = 1.88, *P =* .072 ^b^

*Abbreviations:* Glu, Glutamate; Glx, Glutamate + Glutamine; NAA, N-acetylaspartate; Cho, Choline; ml, myo-Inositol; Cre, Creatine.

^a^Choline data for 2 CHR subjects (one from each of the CBD and placebo groups) was omitted due to CRLB>20%.

^b^Linear contrast accounting for unequal variances. *significant at *P* < .05 level.

### Hippocampal Glutamate – Between-Group Linear Analyses

In our primary a priori analyses, we found a significant linear relationship across groups, such that hippocampal glutamate was highest in healthy controls, lowest in placebo-treated patients, and intermediate in patients treated with CBD (ANOVA unweighted linear term *F*(1,49) = 4.91, *P* = .031, *d* = 0.74) (figure 2A, table 3).

### Glutamate × CBF × Group Interactions

There was a significant interaction between group (control vs placebo), hippocampal glutamate, and CBF in a cluster spanning the left putamen and insula (peak MNI coordinates X/Y/Z = −38/−12/10, T(24) = 5.63, *k* = 679, *P*_FWE_ = .012). Post hoc analysis demonstrated that this was characterized by a strong positive correlation in the placebo group (*r* = 0.68, *P* = .008, *n* = 14) vs a negative correlation in healthy controls (*r* = −0.51, *P* = .027, *n* = 19) (figure 2B). There were no significant interactions in the CBD vs placebo contrast nor the 3-group linear analyses.

### Exploratory Effects on Other Metabolite Levels

Analysis of the secondary/exploratory metabolites revealed significantly lower myo-inositol in the placebo (M ± SD= 5.86 ± 1.01) relative to the control (6.69 ± 1.01) group (*t*(34)=2.46, *P* = .019, *d* = 0.82). Although the direct pairwise CBD (6.09 ± 1.45) vs placebo comparison for myo-inositol was not significant (*P* = .59, *d* = 0.18), there was a significant linear relationship across groups, such that it was highest in healthy controls, lowest in placebo patients and intermediate in patients treated with CBD (ANOVA unweighted linear term *F*(1,49) = 4.58, *P* = .037, *d* = 0.72). There were no other significant effects in pairwise or linear contrast analyses for the other metabolites (Glx, N-acetylaspartate, choline, and creatine)(table 3). Note that these results were not corrected for multiple testing as they are exploratory outcomes only.

## Discussion

This is the first study to investigate the effects of CBD on hippocampal neurochemistry—and its association with regional cerebral perfusion—in people at CHR for psychosis. We first established that hippocampal glutamate levels are lower in CHR patients under placebo relative to healthy controls. To examine our primary hypothesis that CBD would at least partially normalize any such glutamatergic alterations, we then tested for a linear relationship across groups. In line with our predictions, our first key finding was that hippocampal glutamate levels in the CBD-treated CHR group were significantly intermediate between those observed in the placebo group and healthy controls. Finally, we provide the first in vivo evidence that hippocampal glutamate is abnormally associated with perfusion in the striatum and insula in CHR patients relative to controls. Together, these results provide novel insights into the neurobiological mechanisms underlying psychosis risk and suggest that CBD may partially normalize glutamatergic dysfunction in these patients.

### Lower Hippocampal Glutamate in CHR vs Controls

Our finding that hippocampal glutamate was lower in the CHR-placebo group vs controls is consistent with several previous studies demonstrating lower hippocampal Glx^19^ and glutamate^18^ (with additional findings at trend-level)^19,60^ in these patients, as well as negative associations between hippocampal glutamate, striatal dopamine, and symptom severity, particularly in those who go on to transition^61^ (although see^62^). However, other studies have found increased hippocampal Glx^17^ (also in those with genetic risk,^63^ but not always^64,65^), no differences,^20–23,62,66^ or differences only *within* CHR patients based on poor vs good clinical outcomes.^23^ The reasons for the disparity in the presence and/or direction of results are unclear, but sample characteristics^67^ or methodological factors such as voxel location, metabolite correction (for voxel tissue content vs creatine-scaled), as well as the heterogeneity inherent within CHR populations^23,68,69^ may contribute. These findings are compatible with the several meta-analyses that have synthesized this literature,^6,24,25^ which report numerically (but nonsignificantly) lower hippocampal glutamate levels in CHR individuals relative to controls (SMD[g] = −0.26, 95% CI: −0.56 to 0.04, *P* = .09).^25^

On the other hand, our findings are somewhat at odds with preclinical circuit models of psychosis onset, which propose that NMDA receptor hypofunction ultimately leads to excess hippocampal glutamate.^2,3^ Numerous studies in patients with established psychosis^7,14–16^ (and at least one in CHR^17^) have corroborated this pattern of elevated glutamatergic metabolites, but it is noteworthy that several recent meta- and mega-analyses found no significant differences (in hippocampal/mediotemporal regions) in patients across the psychosis continuum relative to controls.^24,25,70^ One potential factor which may account for the differential findings in animals vs humans is that while extracellular glutamate can be measured proximally with invasive methods in rodents,^3^ clinical ^1^H-MRS studies at 3T require a large voxel size and the signal reflects intracellular as well extracellular glutamate (and glutamine) involved in both neurotransmission and metabolism.^71^ Interestingly, studies examining hippocampal Glx (rather than glutamate) appear to find more consistent increases in patients, with meta-analytic effect sizes^24,25^ tending to show numerically (albeit not significantly) increased Glx in CHR relative to controls (SMD[g] = 0.13, 95% CI: −0.43 to 0.69, *P* = .66),^25^ which contrasts with the aforementioned meta-analytic findings for glutamate. This may suggest that glutamine is contributing strongly to the observed increases in ^1^H-MRS-derived metabolite levels and potentially implicates aberrant glutamate-glutamine cycling.^72^ Higher MRI field strengths and advanced techniques (such as GluCEST and ^13^C-MRS) are now becoming available and will enable future research to more reliably separate spectral components and further address these questions.^73,74^

### CBD may Increase Hippocampal Glutamate in CHR

Our first key finding was that CHR patients treated with a single dose of CBD show intermediate levels of hippocampal glutamate relative to controls and patients under placebo. Although our study was cross-sectional with parallel groups, these results suggest that a single dose of CBD may partially normalize the altered glutamate levels we observed in CHR patients. Supporting this view, in our previous within-subject study in people with first-episode psychosis, we showed that a 600 mg dose of CBD significantly increased hippocampal glutamate relative to placebo.^53^ Moreover, CBD was associated with a significantly greater decrease in symptom severity, and a significant inverse relationship was found between hippocampal glutamate and the severity of psychotic symptoms posttreatment.^53^ This suggests that the antipsychotic effects of CBD in patients with psychosis^42,43^ may be related to the increase in hippocampal glutamate observed under its influence.^53^ Outside of the hippocampus, CBD increases Glx in basal ganglia but reduces Glx in prefrontal cortex across ASD and neurotypical individuals.^54^ Preclinical studies demonstrate that CBD can increase prefrontal glutamate in depression models,^75^ although attenuated hippocampal glutamate release has been observed in seizure models.^76^ CBD may also act on excitation-inhibition balance via the GABAergic system^77^ (and see Supplement for discussion of anxiety^78^). In humans, CBD increases GABA in basal ganglia and prefrontal cortex in controls, but decreases GABA in these regions in ASD individuals.^54^ Overall, previous work points to effects of CBD on the glutamate system but the regions implicated and the direction of effects are somewhat mixed, potentially due to species-specific differences and the differential populations examined in humans. Our results therefore extend the limited body of existing literature (so far conducted in people with established psychosis, ASD, and neurotypical controls) by showing that CBD may also modulate hippocampal glutamate in people at risk of psychosis, and in a direction indicative of normalization. However, it should be noted that we did not find significant differences in the CBD vs placebo pairwise analysis. This may be due to the relatively modest sample sizes (*n* = 17/16 per CHR group) and thus limited power for detecting effects of smaller magnitude (see supplementary material for post hoc power calculations). This lack of differences is perhaps unsurprising, since it would be unlikely that a single dose of CBD would fully normalize glutamatergic dysfunction in CHR patients. Therefore, with this in mind, we tested our hypothesis of a partial normalization effect of CBD directly using the 3-way linear analyses, as in our previous publications in this sample.^51,52,59^ Our findings lend support to the idea that CBD may hold value as a potential therapeutic avenue to be pursued in further clinical studies.

### Abnormal Relationship Between Glutamate and Perfusion in CHR

In exploring the broader mechanistic relevance of glutamatergic dysfunction, our second key—and novel—finding was of a significant group (control vs placebo) × glutamate × CBF interaction in a putamen-insula cluster, driven by a strong positive association in the CHR-placebo group and a negative association in controls. Dysfunctional relationships between hippocampal and putaminal physiology are of particular interest as the striatum is a key node in circuit-based models of psychosis.^1^ These propose that deficits in hippocampal inhibition lead to excess excitatory drive in projections to the midbrain-striatum, striatal hyperdopaminergia, and the emergence of psychotic symptoms.^1,4,5^ If, as our results in healthy controls suggest, the normative relationship is such that greater hippocampal glutamate is associated with lower striatal CBF, the positive association we observed in CHR patients could reflect a disruption in inhibitory/homeostatic mechanisms within this hippocampal-midbrain-striatal circuit (supplementary figure S1). Our findings are thus broadly consistent with the aforementioned preclinical models.

In addition, our finding that glutamate was atypically related to perfusion in a cluster localized to the putamen-insula is interesting in light of the putamen hyperperfusion documented in 2 previous CHR studies.^8,27^ Increased putamen CBF in CHR patients^27^ also correlates with positive symptom severity, and lower striatal CBF at follow-up has been associated with greater longitudinal decreases in positive symptoms.^8^ Greater perfusion in the putamen has also been identified as a potential marker of genetic susceptibility for schizophrenia spectrum disorders.^79^ In terms of the insula, perfusion abnormalities have not been definitively reported here in the CHR state, but a recent meta-analysis found conjoint reductions in CBF and glucose metabolism (indexing aberrant neurovascular coupling) within frontoinsular cortex in schizophrenia.^80^ In our previous study in the same sample, we focused exclusively on perfusion and found (during exploratory whole-brain analyses) significantly increased CBF in placebo-treated patients vs controls. This large cluster extended into the left putamen (but not the insula) and partially overlaps with the cluster found here.^50^ The present findings therefore extend our prior work to collectively suggest that (1) hippocampal glutamate is lower, (2) CBF in (clusters spanning) the striatum is greater,^50^ and (3) that the relationship between hippocampal glutamate and striatal-insular perfusion is abnormal in CHR patients relative to controls. While previous work has found CHR-associated dysfunction in the relationship between prefrontal GABA and hippocampal blood flow,^28^ and between hippocampal glutamatergic metabolites and (i) striatal dopamine,^61^ (ii) hippocampal activation (by clinical outcomes),^81^ and (iii) hippocampal-striatal connectivity,^81^ the current study is the first to demonstrate an aberrant relationship between *hippocampal glutamate* and *striatal blood flow* in these patients. Given that hippocampal glutamatergic dysfunction is thought to drive hyperperfusion in the hippocampal-midbrain-striatal circuit, our results provide new empirical evidence of a potential link between these two pathophysiological features in CHR patients, which have so far only been reported in isolation. Our findings therefore provide novel insights on potential mechanisms underlying psychosis risk from a complementary angle to previous literature, and provide a starting point for future research to unpack the nature of these alterations on a more granular level.

## Limitations

Several potential limitations warrant consideration. First, although we found commensurate results in our previous within-subject study,^53^ our parallel-group design means that any effects of CBD should be interpreted with caution. Future within-subject studies would address this issue. Second, CBF data were missing for several CHR subjects, impacting the statistical power of the combined glutamate-CBF analyses. As such, our group × glutamate-CBF results should be considered as initial evidence for hypothesis generation and confirmed by future studies. Post hoc power calculations (supplementary material) also showed that for the glutamate analyses, effect sizes would have to be ~*d* = 1.0 to be detected with the current sample size. Therefore, CBD may have had effects of smaller magnitude but we were unable to detect them. We also administered a single dose of CBD and it remains possible that repeated dosing would produce detectable effects at the current sample size. Going forward, future CBD studies should adopt crossover designs to ensure sufficient power while the number of CHR patients undergoing pharmaco-MRI remains feasible. Finally, ~42% of CHR participants were current cannabis users. Although all were abstinent for >96 hours and none were cannabis dependent, it is possible that the effects of CBD may differ in users vs non-users. However, given that cannabis use is common in CHR cohorts^82^ (particularly in South London),^83^ selecting only non-users may have resulted in an unusual and non-generalizable sample. We therefore recruited a representative CHR sample as typically found in UK services.^84,85^ Post hoc analyses (supplementary material) also suggested that our results were unlikely to be driven by cannabis use, although we were unable to fully examine this for the placebo vs control contrast. Stratification by cannabis use and other potentially important factors—such as the three subgroups of the Comprehensive Assessment of At-Risk Mental States—should therefore be explored in future studies.

## Conclusion

In summary, we found that hippocampal glutamate is lower in CHR patients and may be partially normalized by a single dose of CBD. Furthermore, we provide the first in vivo evidence of an abnormal relationship between hippocampal glutamate and resting perfusion in the striatum and insula in this patient group. Together, these results provide novel insights into the neurobiological mechanisms underlying psychosis risk and suggest that CBD warrants further investigation as a candidate novel treatment.

## Supplementary Material

sgad022_suppl_Supplementary_Material

## Pre-print

A pre-print version of this manuscript was deposited on the medRxiv pre-print server: https://doi.org/10.1101/2023.03.02.23286709.

## Open Access

For the purposes of open access, the author has applied a Creative Commons Attribution (CC BY) license to any Accepted Author Manuscript version arising from this submission.
