# Supplementary material for: Hippocampal Glutamate, Resting Perfusion and the Effects of Cannabidiol in Psychosis Risk
Source: Schizophr Bull Open. 2023 Aug 14;4(1):sgad022. doi: 10.1093/schizbullopen/sgad022 (PMC11207663; doi:10.1093/schizbullopen/sgad022)
Supplement: sgad022_suppl_Supplementary_Material [file sgad022_suppl_Supplementary_Material.pdf]

Hippocampal Glutamate, Resting Perfusion and the Effects of Cannabidiol in Psychosis Risk

Cathy Davies, Matthijs G. Bossong, Daniel Martins, Robin Wilson, Elizabeth Appiah-Kusi, Grace Blest-Hopley, Paul Allen, Fernando Zelaya, David J. Lythgoe, Michael Brammer, Jesus Perez, Philip McGuire, Sagnik Bhattacharyya

SUPPLEMENTARY MATERIAL

Supplementary Introduction

- Fig S1. Schematic of neural circuit mechanisms of hippocampal dysfunction in the pathophysiology underlying psychosis onset

Supplementary Methods

- Participants
- Design, Materials, Procedure
- ^{1}H-MRS Data Processing
- Arterial Spin Labelling Acquisition & Processing

Supplementary Results

- Power Calculations
- Effects of Sex and Cannabis Use on Group Differences in Hippocampal Glutamate

Supplementary Discussion

FIGURE S1. Schematic of proposed neural circuit mechanisms of hippocampal dysfunction in the pathophysiology underlying psychosis onset. In (1), low glutamate signal/input from hypofunctioning NMDARs (akin to faulty homeostatic sensors) prompts GABAergic interneurons to homeostatically increase excitation by reducing inhibition (disinhibition) of glutamatergic pyramidal cells. However, by disinhibiting pyramidal cells (and thus increasing glutamate signalling) in this dysfunctional neural environment, the potential homeostatic adaptation becomes allostatic, with enhanced excitatory drive inducing (2) hypermetabolism and hyperperfusion (elevated blood flow to meet increased metabolic demand), and (3) an overdrive in the responsivity of midbrain dopamine neurons, which project to the associative striatum. Note that the connection between hippocampal pyramidal cells and midbrain dopamine neurons is presented as monosynaptic but is in fact polysynaptic via the ventral striatum and ventral pallidum. Completing the (simplified) circuit, local glutamatergic tone is increased in (4) but is not detected as such by hypofunctioning NMDARs on GABAergic interneurons. Figure reproduced and adapted with permission (CCBY 4.0) from[1]. For original diagrams and discussion of evidence for this proposed circuit, see[2–6]. *Abbreviations:* Glu, glutamate; NMDAR, N-methyl-D-aspartate receptor; CA1, Cornu Ammonis 1.

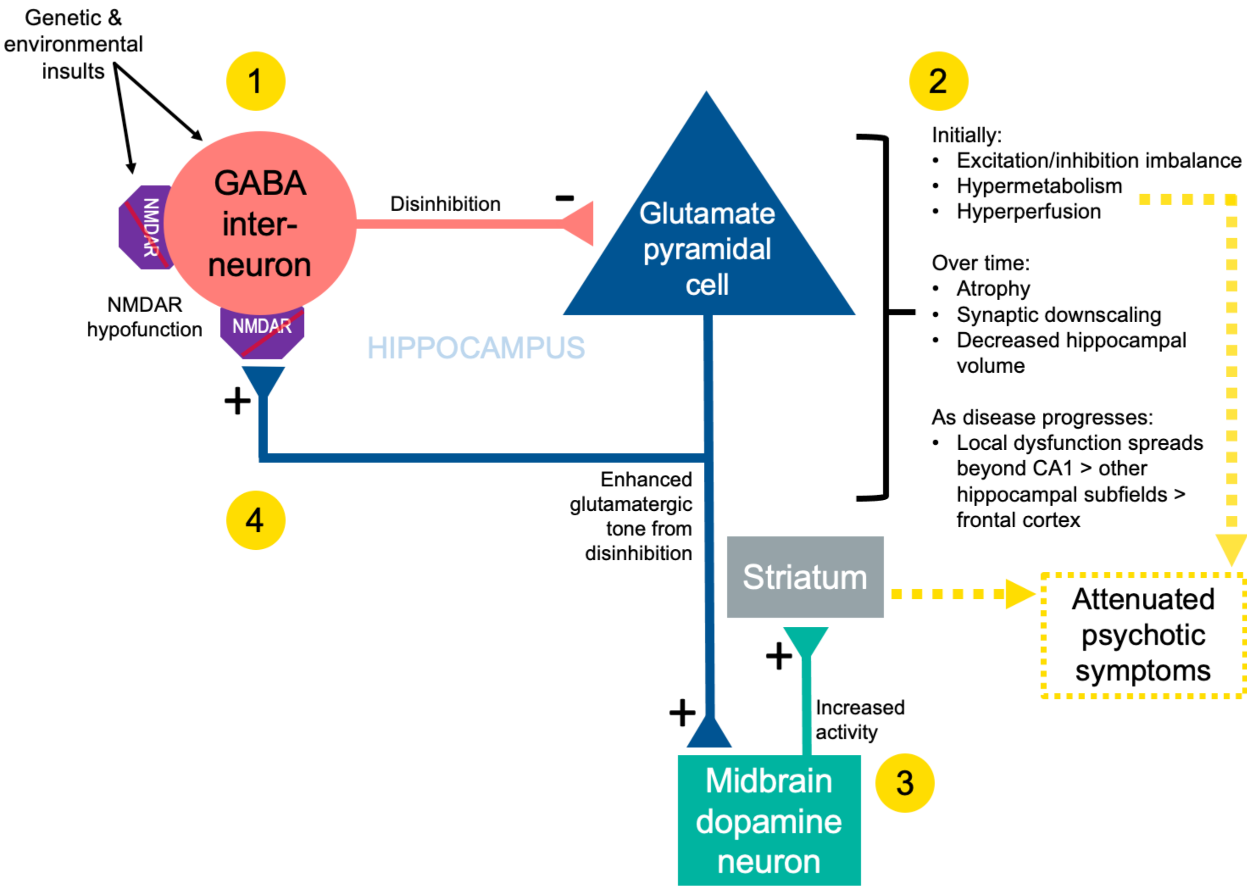

SUPPLEMENTARY METHODS

Participants

Thirty-three antipsychotic-naive CHR individuals, aged 18–35, were recruited from specialist early detection services in the United Kingdom. CHR status was determined using the Comprehensive Assessment of At-Risk Mental States (CAARMS) criteria.[7] Briefly, subjects met one or more of the following subgroup criteria: (a) attenuated psychotic symptoms, (b) brief limited intermittent psychotic symptoms (BLIPS, psychotic episode lasting <1 week, remitting without treatment), or (c) either schizotypal personality disorder or first-degree relative with psychosis, all coupled with functional decline.[7] Participants were required to abstain from cannabis for 96h, other recreational substances for 2 weeks, alcohol for 24h and caffeine and nicotine for 6h before attending. A 96-hour abstinence period was considered sufficient to rule out any acute effects of cannabis intoxication and withdrawal, as previous evidence suggests that mean plasma elimination half-life of THC is around 21.5 hours[8] and most withdrawal symptoms last for around 4 days.[9]

Design, Materials, Procedure

The 600mg dose of CBD was selected based on previous findings that doses of 600-800 mg/day are effective in established psychosis[10] and anxiety.[11] The 180 min interval between drug administration and MRI acquisition was selected based on previous findings describing peak plasma concentrations at 180 min following oral administration.[12,13]

^{1}H-MRS Data Processing

Spectra were analysed using LCModel/6.3-0A[14] using the standard basis set of 16 metabolites (L-alanine, aspartate, creatine, phosphocreatine, GABA, glucose, glutamine, glutamate, glycerophosphocholine [choline], glycine, myo-inositol, L-lactate, N-acetylaspartate, N-acetylaspartylglutamate, phosphocholine, and taurine) acquired at the same field strength (3T), localisation sequence (PRESS), and echo time (30ms) as the ^{1}H-MRS spectra in the current study. Model metabolites and concentrations used in the basis set are detailed in the LCModel manual (http://s-provencher.com/pub/LCModel/manual/manual.pdf).

We calculated and corrected for ^{1}H-MRS voxel tissue content using SPM8 and in-house scripts to (a) segment the T1-weighted structural images into grey matter, white matter, and cerebrospinal fluid (CSF) using SPM8 in Matlab R2017a, (b) locate and map the coordinates of each voxel to the segmented T1 images, and (c) provide the tissue content proportions. Metabolite values were corrected for voxel tissue content using the formula: $M_{corr}= M\times([GM\times1.21] +WM+ [CSF\times1.55]) / (WM+GM)$, where M is the uncorrected metabolite value

and GM/WM/CSF are proportions of grey matter, white matter and CSF, respectively. The formula assumes a CSF water concentration of 55,556 mol/m^3 and the LCModel default brain water concentration of 35,880 mol/m^3.[15,16] Apart from assuming T_2 = 80 ms for tissue water, no corrections were applied for metabolite and water relaxation times.

Arterial Spin Labelling Acquisition & Processing

ASL Image Acquisition

For ASL image registration, a high resolution T2-weighted Fast Spin Echo (FSE) image (TE= 54.58ms, TR= 4380ms, Flip angle= 90deg, FoV= 240, Matrix size= 320 x 320, slice thickness= 2mm, 72 spatial locations) was acquired and used alongside the T1-weighted Spoiled Gradient Recalled (SPGR) images (detailed in the main text). Resting Cerebral Blood Flow (CBF) was measured using 3D pseudo-Continuous Arterial Spin Labelling (CASL) scans acquired with a 3D Fast Spin Echo (FSE) spiral multi-shot readout, following a post-labelling delay of 1.5s. The spiral acquisition used a short (10ms) TE, and 8 spiral arms (interleaves) with 512 points in each arm. FSE TE= 32.26ms, TR = 5500ms. 64 slices of 3mm thickness were obtained and the in-plane FoV was 240×240mm. Three pairs of tagged-untagged images were collected. The whole ASL pulse sequence, including the acquisition of calibration images, was performed in 6:08min.

ASL Image Processing

Data were preprocessed using FMRIB Software Library (FSL) 6.0.2 using the following procedure: (1) T1 and T2 images were skull-stripped and corresponding brain-only binary masks created; (2) original CBF images were coregistered to the T2 images and (3) multiplied by the binary T2 mask to create a skull-stripped CBF image in T2 space; (4) skull-stripped T2 was coregistered to skull-stripped T1; (5) skull-stripped T1 was first linearly coregistered to the MNI152 T1 2mm brain template, before non-linear registration (FNIRT) of the original T1 to MNI space; (6) original T2 images were registered to the MNI template (via T1 space) in a single concatenated step, using the T2-to-T1 transformation matrix (from step 4) and T1-to-MNI warp (from step 5); (7) skull-stripped CBF images (already in T2 space) were registered to the MNI template using the concatenated procedure in step 6; (8) normalised CBF images were spatially smoothed with a 6mm Gaussian kernel. All images were visually inspected for preprocessing errors.

SUPPLEMENTARY RESULTS

Power Calculation

The overarching study (https://doi.org/10.1186/ISRCTN46322781) was primarily powered for fMRI BOLD signal and not for the ^{1}H-MRS or ASL analyses reported here, and thus we did not do a formal power calculation *a priori* based on hippocampal glutamate (original power calculation is available in the supplement of our previous publication in the same sample[17]).

Post-hoc Sensitivity Power Calculations for Hippocampal Glutamate

Given that the original power calculation was not specifically tailored to analyses of glutamate levels, we conducted post-hoc sensitivity power calculations to facilitate consideration of our results in the context of our sample size. Sensitivity power calculations were computed for independent t-tests (two-tailed; 80% power; a=.05) with our sample sizes (CBD=16, placebo=17, controls=19). The associated percent change in glutamate levels for the effect sizes (determined by the sensitivity power calculation) were estimated using the mean and SD of control metabolite values from a previous study by Stone et al[18], and as previously conducted in our earlier work[19] (here for independent t-tests; two-tailed; 80% power; a=.05; assumed SD and sample size equal for both groups).

Post-hoc power calculations suggested that for the placebo vs control group contrast, the minimum effect size (Cohen's d) for a between-group difference in glutamate (detectable at 80% power when a=.05, with our sample size) was d=0.96 (~22.4% difference in glutamate levels).

For the CBD vs placebo group contrast, where we did not find significant results, post-hoc power calculations indicated that the minimum effect size (Cohen's d) for between-group difference in glutamate (detectable at 80% power when a=.05, with our sample size) was d=1.00 (~23.4% difference in glutamate levels). Therefore, it is possible that CBD had (between-group) effects of smaller magnitude but we were unable to detect them with our sample sizes. Future studies with larger samples are therefore needed. It is also worth noting that this study administered only a single acute dose of CBD, and it remains possible that sustained dosing would produce a detectable effect at the current sample size.

Effects of Sex and Cannabis Use on Group Differences in Hippocampal Glutamate

Inclusion of mean-centred sex in an ANCOVA showed that for the placebo vs control contrast, the effect of sex was not significant (F(1,33)= 0.25, p=.62), but the effect of group remained significant (F(1,33)= 5.78, p=.022), with significantly lower glutamate in the placebo relative to the control group: estimated marginal means M (SE) in placebo = 7.44 (0.29) and controls = 8.39 (0.27). Inclusion of mean-centred sex in an ANCOVA for the CBD vs placebo contrast showed no significant effect of sex (F(1,30)= 0.16, p=.70), and the effect of group remained non-significant (F(1,30)= 0.550, p=.46): estimated marginal means M (SE) in the placebo = 7.44 (0.34) and CBD group = 7.81 (0.35).

For the effects of drug (cannabis) use in the placebo vs control contrast, because cannabis partially covaries with group status it was not possible to adjust for this within the analysis. However, as an alternative approach to explore this, we repeated our initial analysis (t-test) restricted to current non-users only, which showed that the placebo vs control difference in hippocampal glutamate was not significant but was in the same direction as our key results (t(27)= 1.76, p=.089) which, given the reduced sample size (n=10 in placebo, n=19 controls), suggests that our results are unlikely to be driven by cannabis use (mean ± SD in non-using [all] controls= 8.41 ± 1.27; and non-using placebo patients= 7.61 ± 0.87). For the CBD vs placebo pairwise contrast, including mean-centred current cannabis use (yes/no) in Quade's nonparametric (due to inhomogeneity of variances) ANCOVA showed no significant group effect after accounting for cannabis use ($F_{[dfh/dfe]}$(1,31)= 1.28, p=.27), which is consistent with the CBD vs placebo pairwise result in the main text.

SUPPLEMENTARY DISCUSSION

Preclinical models show that the hippocampus is a key part of the circuitry mediating anxiety-related behaviours[20] and evidence suggests that both the hippocampus and anxiety may be targeted by CBD and other cannabinoids.[21] In humans, CBD attenuates perfusion and/or activation of mediotemporal and limbic regions in healthy people[22,23] and those with anxiety disorders.[24] CBD also has anxiolytic effects[25,26] in people with social anxiety disorder[11,27] and in healthy people subjected to simulated public speaking.[26,28–30] In our previous work based on the same patient sample as the present study, we showed that 7-day CBD treatment partially attenuated the cortisol and anxiety response to stress in CHR individuals.[31] We also previously showed that a single dose of CBD was sufficient to alter brain activation in CHR patients during fear-processing and in a direction suggestive of normalisation,[32] although in further preliminary analyses, CBD did not ‘restore’ the aberrant relationship between stress-induced cortisol response and mediotemporal activation seen in the CHR-placebo group vs controls.[33] In view of the above, we cannot rule out the possibility that anxiety-related effects may explain or contribute to some of the findings reported in the present study. Future studies relating CBD-induced changes in brain chemistry and/or function with changes in symptoms (including anxiety) would help to disentangle these mechanisms and better characterise relationships with clinical effects.

REFERENCES

1. Davies C, Paloyelis Y, Rutigliano G, et al. Oxytocin modulates hippocampal perfusion in people at clinical high risk for psychosis. *Neuropsychopharmacology*. 2019;44(7):1300-1309. doi:10.1038/s41386-018-0311-6

2. Lisman JE, Coyle JT, Green RW, et al. Circuit-based framework for understanding neurotransmitter and risk gene interactions in schizophrenia. *Trends Neurosci*. 2008;31(5):234-242. doi:10.1016/j.tins.2008.02.005

3. Lieberman JA, Girgis RR, Brucato G, et al. Hippocampal dysfunction in the pathophysiology of schizophrenia: a selective review and hypothesis for early detection and intervention. *Mol Psychiatry*. 2018;23(8):1764-1772. doi:10.1038/mp.2017.249

4. Krystal JH, Anticevic A, Yang GJ, et al. Impaired Tuning of Neural Ensembles and the Pathophysiology of Schizophrenia: A Translational and Computational Neuroscience Perspective. *Biol Psychiatry*. 2017;81(10):874-885. doi:10.1016/j.biopsych.2017.01.004

5. Krystal JH, Anticevic A. Toward Illness Phase-Specific Pharmacotherapy for Schizophrenia. *Biol Psychiatry*. 2015;78(11):738-740. doi:10.1016/j.biopsych.2015.08.017

6. Modinos G, Allen P, Grace AA, McGuire P. Translating the MAM model of psychosis to humans. *Trends Neurosci*. 2015;38(3):129-138. doi:10.1016/j.tins.2014.12.005

7. Yung AR, Pan Yuen H, McGorry PD, et al. Mapping the Onset of Psychosis: The Comprehensive Assessment of At-Risk Mental States. *Aust N Z J Psychiatry*. 2005;39(11-12):964-971. doi:10.1080/j.1440-1614.2005.01714.x

8. Heuberger JAAC, Guan Z, Oyetayo OO, et al. Population Pharmacokinetic Model of THC Integrates Oral, Intravenous, and Pulmonary Dosing and Characterizes Short- and Long-term Pharmacokinetics. *Clin Pharmacokinet*. 2015;54(2):209-219. doi:10.1007/s40262-014-0195-5

9. Budney AJ, Moore BA, Vandrey RG, Hughes JR. The time course and significance of cannabis withdrawal. *J Abnorm Psychol*. 2003;112(3):393. doi:10.1037/0021-843X.112.3.393

10. Leweke FM, Piomelli D, Pahlisch F, et al. Cannabidiol enhances anandamide signaling and alleviates psychotic symptoms of schizophrenia. *Transl Psychiatry*. 2012;2(3):e94-e94. doi:10.1038/tp.2012.15

11. Bergamaschi MM, Queiroz RHC, Chagas MHN, et al. Cannabidiol reduces the anxiety induced by simulated public speaking in treatment-nave social phobia patients. *Neuropsychopharmacology*. 2011;36(6):1219-1226. doi:10.1038/npp.2011.6

12. Millar SA, Stone NL, Yates AS, O'Sullivan SE. A systematic review on the pharmacokinetics of cannabidiol in humans. *Front Pharmacol*. 2018;9(NOV). doi:10.3389/fphar.2018.01365

13. Martin-Santos R, A. Crippa J, Batalla A, et al. Acute Effects of a Single, Oral dose of d9-tetrahydrocannabinol (THC) and Cannabidiol (CBD) Administration in Healthy Volunteers. *Curr Pharm Des*. 2012;18(32):4966-4979. doi:10.2174/138161212802884780

14. Provencher SW. Estimation of metabolite concentrations from localized in vivo proton NMR spectra. *Magn Reson Med Off J Soc Magn Reson Med Soc Magn Reson Med*. 1993;30(6):672-679. doi:10.1002/mrm.1910300604

15. Gasparovic C, Song T, Devier D, et al. Use of tissue water as a concentration reference for proton spectroscopic imaging. *Magn Reson Med*. 2006;55(6):1219-1226. doi:10.1002/mrm.20901

16. Kreis R, Ernst T, Ross BD. Absolute Quantitation of Water and Metabolites in the Human Brain. II. Metabolite Concentrations. *J Magn Reson B*. 1993;102(1):9-19. doi:10.1006/jmrb.1993.1056

17. Bhattacharyya S, Wilson R, Appiah-Kusi E, et al. Effect of Cannabidiol on Medial Temporal, Midbrain, and Striatal Dysfunction in People at Clinical High Risk of Psychosis: A Randomized Clinical Trial. *JAMA Psychiatry*. 2018;75(11):1107-1117. doi:10.1001/jamapsychiatry.2018.2309

18. Stone JM, Day F, Tsagaraki H, et al. Glutamate Dysfunction in People with Prodromal Symptoms of Psychosis: Relationship to Gray Matter Volume. *Biol Psychiatry*. 2009;66(6):533-539. doi:10.1016/j.biopsych.2009.05.006

19. Davies C, Rutigliano G, De Micheli A, et al. Neurochemical effects of oxytocin in people at clinical high risk for psychosis. *Eur Neuropsychopharmacol*. 2019;29(5):601-615. doi:10.1016/j.euroneuro.2019.03.008

20. Xia F, Kheirbek MA. Circuit-Based Biomarkers for Mood and Anxiety Disorders. *Trends Neurosci*. 2020;43(11):902-915. doi:10.1016/j.tins.2020.08.004

21. Patel S, Hill MN, Cheer JF, Wotjak CT, Holmes A. The endocannabinoid system as a target for novel anxiolytic drugs. *Neurosci Biobehav Rev*. 2017;76:56-66. doi:10.1016/j.neubiorev.2016.12.033

22. Crippa JADS, Zuardi AW, Garrido GEJ, et al. Effects of cannabidiol (CBD) on regional cerebral blood flow. *Neuropsychopharmacol Off Publ Am Coll Neuropsychopharmacol*. 2004;29(2):417-426. doi:10.1038/sj.npp.1300340

23. Fusar-Poli P, Crippa JA, Bhattacharyya S, et al. Distinct Effects of Δ9-Tetrahydrocannabinol and Cannabidiol on Neural Activation During Emotional Processing. *Arch Gen Psychiatry*. 2009;66(1):95. doi:10.1001/archgenpsychiatry.2008.519

24. Crippa JAS, Nogueira Derenusson G, Borduqui Ferrari T, et al. Neural basis of anxiolytic effects of cannabidiol (CBD) in generalized social anxiety disorder: A preliminary report. *J Psychopharmacol (Oxf)*. 2011;25(1):121-130. doi:10.1177/0269881110379283

25. Zuardi A, Crippa J, Hallak J, et al. A Critical Review of the Antipsychotic Effects of Cannabidiol: 30 Years of a Translational Investigation. *Curr Pharm Des*. 2012;18(32):5131-5140. doi:10.2174/138161212802884681

26. Crippa JA, Guimarães FS, Campos AC, Zuardi AW. Translational Investigation of the Therapeutic Potential of Cannabidiol (CBD): Toward a New Age. *Front Immunol*. 2018;9(September):1-16. doi:10.3389/fimmu.2018.02009

27. Masataka N. Anxiolytic Effects of Repeated Cannabidiol Treatment in Teenagers With Social Anxiety Disorders. *Front Psychol*. 2019;10(November). doi:10.3389/fpsyg.2019.02466

28. Linares IM, Zuardi AW, Pereira LC, et al. Cannabidiol presents an inverted U-shaped dose-response curve in a simulated public speaking test. *Rev Bras Psiquiatr*. 2019;41(1):9-14. doi:10.1590/1516-4446-2017-0015

29. Zuardi AW, Rodrigues NP, Silva AL, et al. Inverted U-shaped dose-response curve of the anxiolytic effect of cannabidiol during public speaking in real life. *Front Pharmacol*. 2017;8(MAY):1-9. doi:10.3389/fphar.2017.00259

30. Zuardi AW, Cosme RA, Graeff FG, Guimarães FS. Effects of ipsapirone and cannabidiol on human experimental anxiety. *J Psychopharmacol (Oxf)*. 1993;7(1_suppl):82-88. doi:10.1177/026988119300700112

31. Appiah-Kusi E, Petros N, Wilson R, et al. Effects of short-term cannabidiol treatment on response to social stress in subjects at clinical high risk of developing psychosis. *Psychopharmacology (Berl)*. 2020;237(4):1121-1130. doi:10.1007/s00213-019-05442-6

32. Davies C, Wilson R, Appiah-Kusi E, et al. A single dose of cannabidiol modulates medial temporal and striatal function during fear processing in people at clinical high risk for psychosis. *Transl Psychiatry*. 2020;10(1):311. doi:10.1038/s41398-020-0862-2

33. Davies C, Appiah-Kusi E, Wilson R, et al. Altered relationship between cortisol response to social stress and mediotemporal function during fear processing in people at clinical high risk for psychosis: a preliminary report. *Eur Arch Psychiatry Clin Neurosci*. 2021;(0123456789). doi:10.1007/s00406-021-01318-z
